# Displacements of the Uterus

**Published:** 1889-03

**Authors:** 


*Displacements of the Uterus.* By Dr. B. S. SCHULTZE. Translated from the German by JAMESON J. MACAN, M.R.C.S., and Edited by ARTHUR V. MACAN, M.B. Pp. 378. London: H. K. LEWIS. 1888.

We gladly welcome this excellent translation of Schultze's valuable and original work. As a scientific and exhaustive exposition of the pathology and treatment of uterine displacements the work stands alone and unrivalled. It is abundantly and excellently illustrated with *original* woodcuts; it is admirably printed and got up; and in every respect appears, in its English dress, as the worthy and even flattering counterfeit of its German original.

